# Radiotherapy and immunotherapy in cancer treatment: mechanisms of clinical synergy

**DOI:** 10.1172/JCI207550

**Published:** 2026-08-17

**Authors:** Lu Lu, Liufu Deng

**Affiliations:** Shanghai Frontiers Science Center of Drug Target Identification and Delivery, State Key Laboratory of Innovative Immunotherapy, School of Pharmaceutical Sciences, Shanghai Jiao Tong University, Shanghai, China.

## Abstract

Synergizing radiotherapy (RT) with immune checkpoint inhibitors has emerged as a promising strategy for solid tumors. RT acts as a potent immunomodulator, capable of functioning as an in situ vaccine through the induction of immunogenic cell death and activation of innate immune sensing, thereby promoting DC maturation and CD8^+^ T cell responses. However, RT also triggers counter-regulatory immunosuppression, including PD-L1 upregulation and the recruitment of suppressive cells, providing the biological rationale for synergy. Here, we systematically review advances in radioimmunotherapy, covering immunomodulatory mechanisms, clinical optimization of dose and sequencing, and the emerging role of artificial intelligence (AI) in guiding treatment paradigms. We adopt a spatial interaction–centric perspective to synthesize current knowledge on how RT governs the DC/CD8^+^ T cell interaction axis across the tumor microenvironment and tumor-draining lymph nodes, aiming to chart a rational course from empirical combination toward personalized, precision radioimmunotherapy. Furthermore, we explore how AI-driven analysis of radiomics and multiomics data is being applied to predict responders and personalize treatment planning.

## Introduction

Radiotherapy (RT) has been applied in clinical cancer treatment for over a century, utilized in approximately 50% of all cancer treatment regimens with the primary goal of local tumor control (1, 2). Modern RT has been broadly classified into external beam RT, including photon therapy (x-rays/gamma rays) and particle therapy (protons, heavy ions); internal radiation (brachytherapy); and radiopharmaceuticals (e.g., untargeted ¹³¹I or targeted ¹^77^Lu prostate-specific membrane antigen) (3). External beam RT is mostly delivered as high-energy x-rays generated by linear accelerators and represents the dominant modality in clinical oncology and the primary focus of this Review. Conventional fractionation radiotherapy (CFRT) schemes deliver 1.8–2.2 Gy per fraction, whereas modern stereotactic body radiotherapy (SBRT) concentrates 8–24 Gy per fraction into abbreviated courses, exploiting precision to maximize tumoricidal effect while sparing adjacent normal tissue (4).

The recognition of the abscopal effect —regression of distant, nonirradiated lesions after local RT — provided early clinical evidence that RT could elicit a systemic immune response (5–7). However, RT inherently operates as a double-edged sword by simultaneously inducing systemic immunological shifts that can facilitate distant metastasis (8). In reality, the abscopal effect remains rare in clinical practice, indicating that the immune response induced by RT alone is often insufficient to overcome the immunosuppressive tumor microenvironment (TME). These limitations underscore the pressing need for therapeutic strategies that can amplify RT-induced immune responses to achieve durable systemic antitumor efficacy.

The advent of immunotherapy, particularly the development of immune checkpoint inhibitors (ICIs) targeting the CTLA-4 and PD-1/PD-L1 axes, has revolutionized cancer treatment, but such approaches are limited by low response rates in immunologically cold tumors (9–16). This unmet clinical need has catalyzed a paradigm shift in the perception of RT from a direct tumor cell–killing approach to a potent systemic immunomodulatory agent. Mechanistically, RT can function as an in situ vaccine by inducing immunogenic cell death (ICD), promoting the release of tumor-associated antigens and activating innate immune signaling, thereby priming a de novo antitumor immune response (17, 18). Thus, RT holds the potential to convert immunologically cold tumors into hot microenvironments more susceptible to ICI therapy (3). Accordingly, combining RT and immunotherapies (including ICIs, adoptive cell therapy, bispecific T cell engagers [BiTEs], engineered cytokines, etc.) has emerged as a promising strategy to harness these biological interactions to achieve profound therapeutic synergy (19, 20). Landmark integrations, such as the use of consolidation ICIs following chemoradiotherapy in locally advanced non–small cell lung cancer (NSCLC), have decisively validated this synergy and redefined global standards of care (21). However, as the field rapidly expands, critical clinical bottlenecks remain. The translation of striking preclinical synergies into consistent survival benefits in phase III clinical trials is frequently hindered by poorly defined optimal dosimetric parameters, the intrinsic immune resistance of cold tumors, and the complex, overlapping risks of severe toxicities, such as radiation-induced and immune-mediated pneumonitis (22). Studies have established that RT-induced antitumor immunity critically depends on type I IFN–driven (IFN-I–driven) DC-mediated recruitment and activation of CD8^+^ T cells (23, 24), positioning DCs as a central relay in the RT/immune axis. While tumor-intrinsic resistance mechanisms, immunosuppressive myeloid populations, and vascular components of the TME contribute to treatment outcomes, deciphering the DC/CD8^+^ T cell axis represents a central challenge.

In this Review, we first dissect the molecular mechanisms underpinning RT-immunotherapy synergy, while emphasizing the paradoxical feedback loops that drive adaptive resistance. Finally, we synthesize the current clinical landscape and highlight the pivotal role of predictive biomarkers, aiming to chart a rational course from empirical, trial-and-error approaches toward the realization of personalized, precision radioimmunotherapy.

## Mechanisms of RT-immunotherapy synergy

### ICD induction and antigen release.

**Figure 1:**
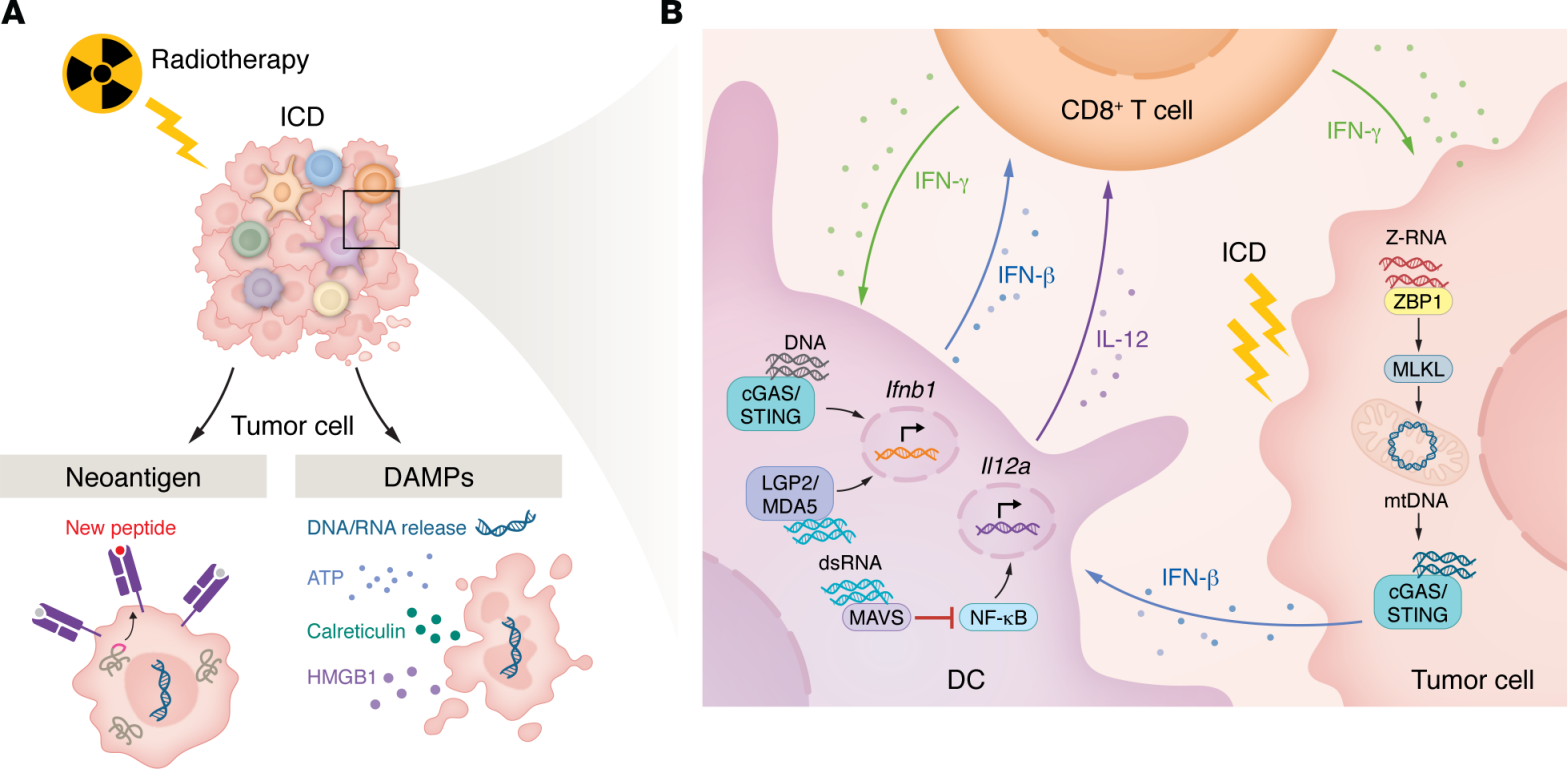
RT-induced innate immune sensing. (**A**) RT functions as an in situ vaccine by inducing ICD, which promotes the release of tumor-associated antigens, neoantigens, and DAMPs, including ATP, calreticulin, and HMGB1. These signals promote DC activation and antigen uptake. (**B**) DC-intrinsic DNA and RNA sensing pathways are indispensable for type I IFN–dependent effective CD8^+^ T cell priming after RT, whereas canonical RIG-I/MAVS signaling in DCs can paradoxically impair cross-priming through inhibiting IL-12 production. ZBP1 further functions as a dual RNA/DNA sensor that detects RT-induced nucleic acids, triggers necroptosis, and amplifies STING-dependent immune activation, thereby reinforcing DC-dependent adaptive antitumor immunity.

RT induces ICD through the spatiotemporally coordinated release of damage-associated molecular patterns (DAMPs; e.g., calreticulin, ATP, dsDNA), which act as critical danger signals to drive DC maturation (Figure 1) (25–29). In addition, RT enhances the release and presentation of tumor antigens through multiple complementary mechanisms, including broadening of the neoantigen repertoire, generation of novel neoantigens via enhanced immunoproteasome activity, increased MHC class I presentation, and RT-induced antigen upregulation, thereby enabling immunotherapy responses even in tumors with low mutational burden and effectively contributing to the in situ vaccine effect (30–36). By providing both tumor-associated antigens and proinflammatory DAMPs, RT-induced ICD matures DCs and licenses their migration to tumor-draining lymph nodes (TDLNs). Within the TDLNs, these mature DCs process and cross-present internalized tumor antigens on MHC class I molecules to naive CD8^+^ T cells, thereby bridging localized tumor damage with the initiation of a systemic adaptive immune response (37). However, it is critical to note that while RT alone effectively initiates this sequence, the T cell response is frequently blunted by the immunosuppressive networks of the TME or the upregulation of the PD-1/PD-L1 axis (38). This biological reality provides the mechanistic rationale for combining RT with ICIs.

### Nucleic acid sensing initiates DC antitumor immunity.

Here, we focus on the initiation of immunity through nucleic acid sensing and the pivotal role of DCs in determining immune outcomes to interpret the core challenge in harnessing DCs for effective radioimmunotherapy. Specifically, RT induces the formation of micronuclei and the accumulation of dsDNA fragments in the cytoplasm of irradiated tumor cells (39). This cytosolic dsDNA is primarily detected by cyclic GMP-AMP synthase (cGAS), though other sensors like Z-DNA binding protein 1 (ZBP1) and DexH-box helicase 58 may also play a role (40). Upon recognition of cytosolic dsDNA, cGAS synthesizes 2′3′-cGAMP from ATP and GTP, thereby activating the stimulator of interferon genes (STING) pathway to drive transcription of IFN-Is and IFN-stimulated genes (41, 42). This RT-induced innate sensing creates durable immune reprogramming. IFN-Is are critical for orchestrating the radiation-induced adaptive immune response, primarily by promoting the recruitment, maturation, and cross-priming capabilities of conventional type 1 DCs (cDC1s) (23, 43).

Accordingly, STING agonists have emerged as a rational approach to potentiate the immune response in cancer immunotherapy. However, despite preclinical promise, STING agonists have largely failed in trials due to delivery and toxicity issues and context-dependent immune effects (44, 45). The progress of STING agonists and the mechanisms of acquired resistance have been systematically reviewed elsewhere (46, 47). The therapeutic exploitation of STING is complicated by its dichotomous role in the TME. While acute STING/IFN-I activation supports DC maturation and CD8^+^ T cell priming, chronic STING activation paradoxically induces immunosuppressive programs, including tolerogenic DC differentiation; upregulation of PD-L1, IDO1, and SerpinB9-mediated resistance; and recruitment of myeloid-derived suppressor cells (MDSCs), thereby undermining the immune responses (48–52). In addition, RT induced IFN-I may act as a regulator of inflammatory memory that shapes long-term antitumor immunity. IFN-I activates the JAK/STAT pathway, which in turn drives histone modifications (e.g., H3K4me3) and chromatin remodeling at cytokine and pattern recognition receptor gene loci in macrophages, NK cells, and DCs (53). These epigenetic changes prime the innate immune system for enhanced responses upon secondary tumor antigen exposure or rechallenge. For example, trained macrophages display sustained upregulation of costimulatory molecules and increased secretion of proinflammatory cytokines (IL-1β, IL-6), thereby enhancing their capacity to prime tumor-specific T cells (54). The induction of trained immunity by certain agonists, including RT-derived signals, is increasingly recognized as a mechanism that contributes to durable antitumor responses, as comprehensively reviewed in recent literature (55).

Furthermore, the activation of the cGAS/STING axis is highly dose dependent, dictated by the expression threshold of the primary cytosolic DNA exonuclease TREX1. Biologically, moderate doses below 10 Gy per fraction appear optimal for inducing ICD and activating the cGAS/STING pathway (56–61). In contrast, high-dose SBRT (>10 Gy per fraction) disrupts tumor vasculature and activates TREX1, which can degrade cytosolic DNA and dampen IFN-I signaling and may limit antitumor immunity (62, 63).

Beyond DNA sensing, RT-induced cytosolic RNA activates RIG-I–like receptor pathways (RIG-I, MDA5, and MAVS) to promote IFN-I production and DC activation (64–66). However, the immunological effects of RNA sensors are highly context dependent. While the RIG-I–like receptor LGP2 supports RT-induced DC enrichment and cross-priming (67, 68), canonical RIG-I/MAVS signaling in DCs may paradoxically suppress these processes (69). In addition, ZBP1 functions as a dual RNA/DNA sensor that mediates RT-induced ICD and subsequent DC-dependent adaptive immunity (70, 71), although its direct roles in DCs and CD8^+^ T cells remain unclear.

Together, these findings underscore the complexity of nucleic acid sensing in shaping RT-driven immune responses, indicating that RT-induced innate sensing is not uniformly immunostimulatory; rather it dynamically regulates the balance between durable antitumor immunity and chronic inflammatory dysfunction.

### Reprogramming the TME.

**Figure 2:**
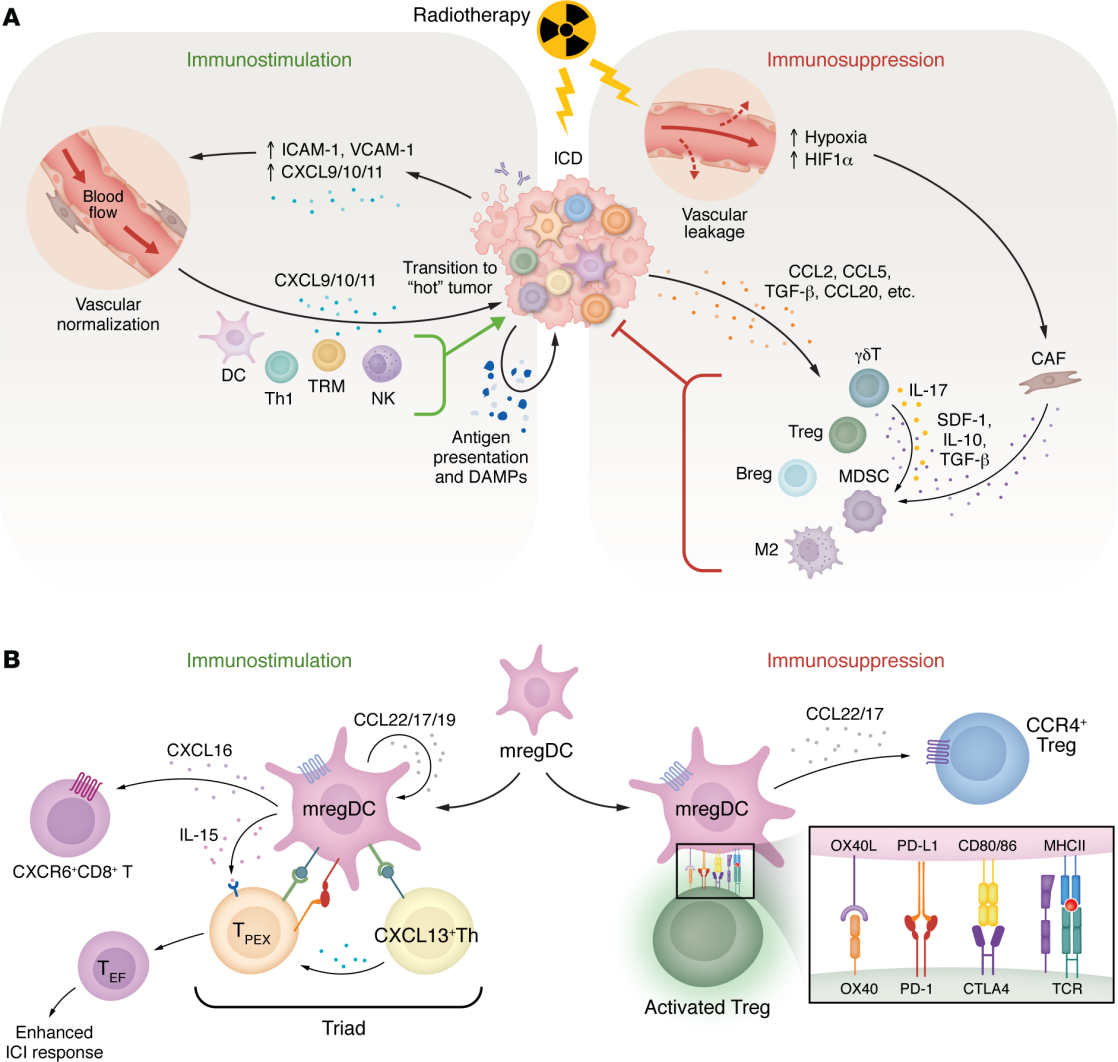
RT-driven TME remodeling. RT reshapes the TME through multiple mechanisms. (**A**) Moderate or fractionated RT leads to vascular normalization by upregulating endothelial adhesion molecules (ICAM-1, VCAM-1, and E-selectin) and chemokine production (CXCL9, CXCL10, and CXCL11) to promote lymphocyte infiltration, thereby enhancing antitumor immunity and facilitating the transition from cold to hot tumors. In contrast, following high ablative doses, radiation-driven cytokines (CCL2, CCL5, TGF-β, etc.) recruit and expand Tregs, MDSCs, M2-polarized macrophages, regulatory B cells (Bregs), and γδ T cells, which dampen CD8^+^ T cell responses via IL-10, arginase-1, and TGF-β. These doses induce vascular damage and hypoxia, activating HIF-1α/SDF-1 signaling to recruit immunosuppressive myeloid populations. (**B**) mregDCs serve as a pivotal hub. By forming cellular triads with CXCL13^+^ helper T cells and T_PEX_ cells, mregDCs promote effector differentiation through IL-15 transpresentation and CXCL16 secretion. In contrast, mregDCs also recruit Tregs via CCL22/CCL17 and engage activated Tregs through antigen presentation and coregulatory receptor–ligand interactions, including MHC II–TCR, OX40L–OX40, PD-L1–PD-1, and CD80/86–CTLA-4, thereby supporting Treg activation and reinforcing local immunosuppression.

Beyond direct induction of DNA damage and ICD, RT fundamentally reshapes the TME through the cellular and cytokine/chemokine environment and through vasculature remodeling (Figure 2). RT-driven inflammatory cytokines and chemokines, including CCL2, CCL5, CSF1, and TGF-β, promote the recruitment or expansion of immunosuppressive cells such as Tregs (72, 73), γδ T cells (74), immunosuppressive regulatory B cells (75), and MDSCs (76). These suppressive populations rapidly dampen the RT-induced CD8^+^ T cell response by secreting IL-10 and arginase-1 and thus represent the major barrier limiting the therapeutic efficacy of RT (77). Moreover, radiation promotes the polarization of tumor-associated macrophages toward an immunosuppressive M2-like phenotype and activates cancer-associated fibroblasts (CAFs), which can physically exclude effector cells and remodel the extracellular matrix (78). RT also exerts immunosuppressive effects by inducing tumor-derived factors, such as amphiregulin, which reprogram EGFR^+^ myeloid cells toward an immunosuppressive phenotype and suppress antitumor phagocytosis, thereby promoting tumor progression and therapeutic resistance (8).

In addition, RT-induced vascular leakiness reshapes the tumor immune microenvironment by facilitating immune cell trafficking into irradiated tumors. Moderate or fractionated RT promotes vascular normalization by upregulating endothelial adhesion molecules (e.g., ICAM-1, VCAM-1, and E-selectin) and inducing an M1-like polarization of perivascular macrophages, thereby alleviating hypoxia and enhancing lymphocyte infiltration into tumors (79). In contrast, high ablative doses or prolonged CFRT can induce severe vascular damage and hypoxia. Activated HIF-1α promotes CAF-derived stromal cell–derived factor-1 secretion, which recruits immunosuppressive myeloid populations, including neutrophils, MDSCs, and proangiogenic TIE2^+^ macrophages, while simultaneously enhancing immune checkpoint signaling through upregulation of PD-L1 on tumor cells and modulation of DC function (80–82). Together, these effects promote vascular restoration, immune evasion, and tumor progression. Therefore, RT-mediated vessel leakiness exerts a dual role in modulating antitumor immunity and represents an important component of the interplay between RT and the immune microenvironment.

### The mregDC axis in RT-induced antitumor immunity.

As noted, RT promotes antitumor immunity by inducing FMS-like tyrosine kinase 3 ligand–mediated (FLT3-L–mediated) DC precursor expansion and CCR7 ligand–dependent trafficking to TDLNs (83–85). Recent single-cell analyses have identified a conserved population of mregDCs (mature DCs enriched in immunoregulatory molecules; CCR7^+^LAMP3^+^) that emerges from cDC1/cDC2 lineages upon tumor antigen capture (86–90). These mregDCs coexpress immunoregulatory molecules (PD-L1/PD-L2/CD200), migration/maturation markers (CCR7/CD40), and proinflammatory cytokines (IL-12/IL-15), positioning them at the interface between innate sensing and adaptive priming (90–93). Notably, mregDC abundance predicts clinical responses to ICIs, and the ratio of mregDCs to TCF1^+^CD8^+^ T cells serves as an independent prognostic factor in melanoma (94).

The effects of mregDCs are context dependent. Functionally, mregDCs facilitate CCR7-mediated migration to lymph nodes and promote the expansion of stem-like or progenitor exhausted (T_PEX_; TCF1^+^PD-1^+^TIM3^–^) CD8^+^ T cells, which retain self-renewal capacity and potential to differentiate to effector CD8^+^ T cells (95–97). While in the TME, mregDCs form cellular triads with CXCL13^+^ T helper cells and PD-1^hi^ progenitor CD8^+^ T cells through CCL19/CCL22/CCL17- and CD80/CD86-mediated costimulation, driving effector differentiation upon PD-1 blockade (95, 98). CCR7^+^ mregDCs also provide IL-15 trans-presentation and CXCL16 secretion to support CXCR6^+^ CD8^+^ T cell responses (99). By contrast, mregDCs recruit Tregs via CCL22/CCL17 to establish immunosuppressive niches (100, 101), a mechanism that has been linked to radioresistance (102). Emerging evidence indicates that microbiota-driven enhancement of CCR7^+^PD-L1^+^ mregDC trafficking can potentiate the efficacy of low-dose irradiation combined with PD-L1 blockade (103). However, the precise roles of mregDCs under RT conditions remain to be fully elucidated. Given that mregDCs exert dual roles in antitumor immunity, overcoming their immunosuppressive functions while reinforcing crosstalk with CD8^+^ T cells represents a promising strategy to enhance immunotherapy efficacy.

### CD8^+^ T cell heterogeneity defines therapeutic responsiveness.

Despite these immunostimulatory effects, RT simultaneously imposes constraints on CD8^+^ T cell immunity. Lymphocytes are highly radiosensitive, and irradiation can induce transient lymphodepletion and disrupt systemic T cell dynamics (104–107). In mice, RT transiently impairs systemic T cell circulation for up to 3 days, but by 5 days following radiation, there is increased T cell recirculation from the TDLNs, indicating rapid recovery of lymphocyte trafficking (108). By inducing local inflammation and IFN-γ production, RT robustly upregulates the secretion of crucial chemokines, particularly CXCL9, CXCL10, and CXCL11, recruiting CXCR3^+^CD8^+^ T cells into the TME (109). Furthermore, RT induces expression of CXCL16, which binds CXCR6 on Th1 and CD8^+^ T cells, effectively converting a barren TME into an inflamed and highly infiltrated microenvironment (109). However, RT preserves and activates tumor-resident memory T cells, which exhibit relative radioresistance and can mediate effective local tumor control independent of newly infiltrating T cells (110). Notably, RT also enhances NK cell cytotoxicity, which also has the potential to promote CD8^+^ T cell responses (111, 112). In the context of RT combined with ICIs, both resident and newly recruited T cells appear to contribute, with optimal therapeutic efficacy often requiring the interplay of both populations (113).

CD8^+^ T cell differentiation states, rather than mere abundance, critically determine responses to RT and immunotherapy. In the canonical cancer-immunity cycle, naive CD8^+^ T cells are primed in TDLNs and then migrate to tumors to kill cancer cells. However, persistent antigen exposure in the TME drives hierarchical CD8^+^ T cell exhaustion (TCF1^–^PD-1^+^TIM3^+^) and epigenetic remodeling (48, 114, 115). Single-cell analyses have revealed extensive heterogeneity among tumor-infiltrating CD8^+^ T cells, identifying distinct TCF-1^+^/CXCL13^+^ T_PEX_, granzyme K–expressing (GZMK^+^) effector/effector memory T, and terminally exhausted states that exhibit differential responses to immune checkpoint blockade (116, 117). T_PEX_ cells have emerged as the principal responders to ICI and adoptive T cell therapies, and their presence strongly correlates with durable clinical benefit (118–123). Fractionated RT combined with ICIs generates long-lived memory CD8^+^ T cell responses that enable complete rejection of tumor rechallenge months after initial treatment (124–126). Consistently, high-dose RT enriches T_PEX_ cells in a cGAS/STING-dependent manner and promotes their proliferation and differentiation into effector progeny (127, 128).

### TDLNs as reservoirs for T_PEX_ cells.

**Figure 3:**
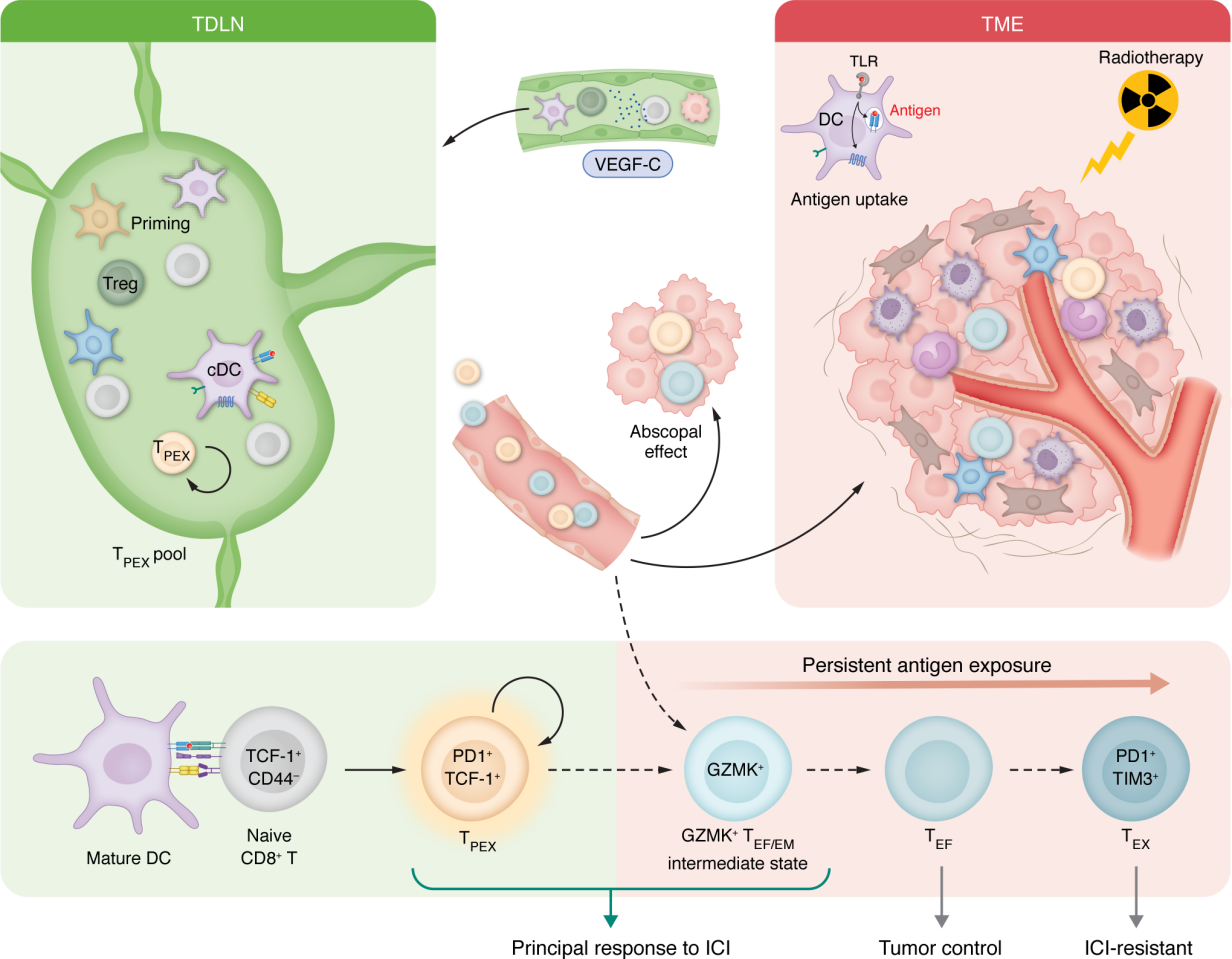
TDLN-orchestrated DC/CD8^+^ T cell immunity. The DC/CD8^+^ T cell axis functions across 2 anatomically and functionally distinct compartments. In TDLNs (left), CCR7^+^ mature DCs from irradiated tumors cross-present tumor antigens on MHC I to naive CD8^+^ T cells. DCs establish specialized niches to generate and maintain TCF1^+^ T_PEX_ cells, the principal drivers of durable antitumor responses. Tregs within TDLNs can suppress priming. In the TME (right), T cells enter and further differentiate upon antigen reencounter to exert an antitumor response, including an abscopal effect. Naive CD8^+^ T cells (TCF1^+^CD44^–^) are primed into T_PEX_ in lymph nodes and then migrate to the TME. Within the TME, GZMK^+^CD8^+^ T cells represent a distinct effector memory–like population and occupy an intermediate position within intratumoral CD8^+^ T cell differentiation. Progenitors are the main responders to ICIs, whereas terminally exhausted cells are largely resistant. T_EF_, effector CD8^+^ T cells; T_EX_, terminally exhausted CD8^+^ T cells.

TDLNs are the principal counterparts for both the generation of tumor-specific immunity and the immunopotentiation effects of cancer immunotherapy (Figure 3) (129–133). TDLNs harbor T_PEX_ cells that retain proliferative capacity and continuously replenish the intratumoral T cell pool (130, 134, 135). These cells migrate into tumors and undergo further differentiation upon antigen re-encounter, thereby sustaining effector responses. RT critically modulates this TDLN/tumor axis. Activation and expansion of T_PEX_ cells within TDLNs has been implicated in the therapeutic synergy between RT and ICI therapy (136). Conversely, irradiation of draining lymph nodes reduces immune infiltration and compromises tumor control, likely through depletion of tumor-specific CD8^+^ T cells and impairment of their maintenance and trafficking (137–139). Thus, preservation or delayed irradiation of TDLNs restores both local and systemic antitumor responses, highlighting the essential role of intact lymphoid niches in sustaining T_PEX_-mediated immunity (139, 140).

However, the immunological consequences of TDLN irradiation are highly context dependent. Standard CFRT protocols routinely involve elective nodal irradiation (ENI), which directly exposes TDLNs to cytotoxic radiation, including clinically uninvolved basins. Preclinical models demonstrate that ENI impairs adaptive immune responses and limits the synergistic benefits of combined radioimmunotherapy (138, 141), highlighting the necessity of preserving lymph node integrity. In contrast, modern involved-node radiotherapy, which confines treatment to radiographically involved lymph nodes only, appears to spare sufficient lymphoid architecture to sustain adaptive immune priming (142–144). However, precisely identifying which lymph nodes harbor occult metastatic disease versus those that remain uninvolved is difficult with current imaging modalities.

The response to TDLN irradiation is also shaped by tumor-specific factors. Cancer cells can reprogram TDLNs into an immunosuppressive state through stromal remodeling and lymph-borne tumor-derived factors, creating a permissive microenvironment that supports both metastatic seeding and immune evasion (145). Consequently, the net impact of nodal irradiation depends on whether TDLNs harbor micrometastatic disease or remain pathologically uninvolved. In head and neck squamous cell carcinoma (HNSCC), ENI has been mechanistically linked to suppressed systemic immunity and compromised ICI response (132). Conversely, in NSCLC, combining dual ICI with neoadjuvant chemoradiotherapy increased the infiltration of activated cytotoxic CD8^+^ T cells and enhanced type I immune responses within irradiated TDLNs even at high cumulative doses (50–60 Gy) (146), suggesting that nodal irradiation may eliminate immunosuppressive tumor niches while preserving — or even enhancing — adaptive immune priming. Collectively, these considerations underscore that the impact of RT on TDLN-mediated immunity is shaped by the interplay of radiation field design, dose fractionation, treatment sequencing, tumor histology, and nodal disease status and warrant a nuanced, indication-specific approach when combining RT with immunotherapy.

## Clinical landscape and challenges of radioimmunotherapy

Preclinical studies have provided robust evidence supporting the synergistic mechanisms between RT and ICI, establishing a strong foundation for their combined use in solid tumors (147). Despite these promising biological rationales, clinical translation of radioimmunotherapy has yielded heterogeneous outcomes in phase III trials. This inconsistency highlights critical gaps in current therapeutic strategies and patient selection criteria.

### From PACIFIC success to failure of concurrent therapies.

The therapeutic potential of combining RT with immunotherapy was firmly established by the PACIFIC trial (ClinicalTrials.gov NCT02125461). This study defined a new standard of care for unresectable stage III NSCLC by demonstrating that consolidation therapy with the ICI durvalumab following chemoradiotherapy confers a statistically significant and durable survival benefit. Long-term follow-up revealed a 5-year overall survival rate of 42.9% in the durvalumab cohort compared with 33.4% in the placebo group (21, 148, 149).

Based on the hypothesis that earlier immune activation could further enhance therapeutic synergy, subsequent research efforts focused on concurrent administration of RT and ICI (150, 151). However, multiple phase III clinical trials investigating this strategy have failed to demonstrate clinical benefits. In NSCLC, the phase III PACIFIC-2 trial (ClinicalTrials.gov NCT03519971) evaluating simultaneous durvalumab and concurrent chemoradiotherapy failed to improve progression-free survival or overall survival (152). Similarly, the CheckMate 73L trial (ClinicalTrials.gov NCT04026412), which investigated concurrent chemoradiotherapy followed by nivolumab with or without ipilimumab, missed its primary progression-free survival endpoint (153). This pattern of clinical failure is also evident in other malignancies. In HNSCC, several phase III trials, including KEYNOTE-412 (ClinicalTrials.gov NCT03040999), also failed to demonstrate a survival benefit when combining PD-1/PD-L1 inhibitors with fractionated concurrent chemoradiotherapy (154–158). Ongoing trials such as EA5181 (ClinicalTrials.gov NCT04092283) are currently facing intense scrutiny regarding concurrent toxicity (159). These results suggest that the temporal interval between the lymphodepleting effects of cytotoxic radiation and the initiation of immunotherapy may influence treatment outcomes.

### Optimal sequencing of RT and immunotherapy.

Mechanistically, radiation creates a transient optimal window for intervention by upregulating checkpoint molecules such as PD-L1 and inducing a time-dependent release of immunological adjuvants (38, 127, 160). Preclinical evidence largely supports administering ICIs concurrently or immediately following RT to capitalize on this peak immune stimulation, whereas prior ICI administration may detrimentally radiosensitize CD8^+^ T cells and abrogate systemic effects (38, 161–163). In clinical practice, however, the ideal timing remains ambiguous and highly tumor specific, with various trials demonstrating efficacy using both sequential and concurrent approaches (148, 164–167). Additionally, an adequate interval may be required to mitigate severe toxicity in the clinic (168). Conversely, an excessive interval between RT and immunotherapy may weaken the synergistic effect (38, 169). Optimal sequencing is also agent specific, as the distinct mechanisms of anti–PD-1/PD-L1 and anti–CTLA-4 inhibitors dictate different ideal timings relative to the RT-induced immune cascade. Specifically, anti–CTLA-4 is optimally administered before RT, as it depletes Tregs and thereby facilitates antigen cross-presentation and T cell priming in the subsequent radiation-induced immune cascade (170, 171). In contrast, anti–PD-1/PD-L1 blockade is most effective when given concurrently with or shortly after RT, capitalizing on the RT-induced upregulation of PD-L1 on tumor cells and infiltrating immune cells to reinvigorate intratumoral exhausted T cells (172). Ultimately, determining the optimal timing requires further investigation, potentially utilizing the real-time monitoring of peripheral T cell expansion and activation after RT to guide personalized immunotherapy initiation.

Overall, current clinical evidence suggests that RT-immunotherapy combinations are generally well tolerated, with manageable toxicity profiles and no substantial increase in severe immune-related adverse events (144). Nevertheless, treatment-related toxicities, particularly pneumonitis, warrant careful monitoring, highlighting the need for more precise and individualized therapeutic strategies.

### RT combined with other immunotherapeutic strategies.

While the combination of RT with ICIs has been the most extensively studied pairing, of additional interest is the integration of RT with more targeted, cell-based, and protein-engineered immunotherapies. RT can improve T cell trafficking, antigen availability, and cross-priming, thereby enhancing the efficacy of CAR T, TCR T, and tumor-infiltrating lymphocyte therapy (173, 174). Clinically, RT has also been used as a bridging strategy before CAR T infusion in hematologic malignancies to achieve cytoreduction and potentially exploit its immunostimulatory effects (175). Furthermore, RT may enhance the efficacy of other immunotherapies such as bispecific antibodies and engineered cytokines. For instance, RT (8–10 Gy) combined with the anti–TGF-β/PD-L1 bispecific antibody YM101 inhibited tumor growth and prolonged survival in preclinical models, with RT dose-dependently promoting DC maturation and increasing intratumoral DC and tumor-infiltrating lymphocyte accumulation, while YM101 simultaneously mitigated radiation-induced pulmonary fibrosis (176). The ability of BiTEs and trispecific targeting chimera strategies to reinvigorate exhausted T cell responses (177–179) is constrained in solid tumors by limited T cell infiltration and insufficient antigen availability (180), and incorporating RT into these regimens may help overcome these barriers. In addition, a recent review systematically delineates the interplay between RT and cytokines, highlighting the spatiotemporal regulation of cytokines such as IL-15, IL-12, and IFNs in the context of RT, as well as emerging combinatorial strategies involving RT-derived microparticles harboring tethered IL-15 (tIL-15) and tethered CCL19 (tCCL19), and PD-1 blockade (181). Importantly, many of these strategies aim to overcome RT-associated immunosuppressive remodeling, including MDSC accumulation, Treg expansion, and CD8^+^ T cell exhaustion, thereby broadening the therapeutic landscape of radioimmunotherapy beyond checkpoint inhibition.

## Future directions and therapeutic optimization

### Immunologically guided hypofractionation and lymph node preservation.

To preserve the TDLN/DC/CD8^+^ T cell axis, the clinical landscape is rapidly shifting from conventional fractionation to immunologically guided hypofractionation, such as SBRT. By utilizing highly conformal techniques to deliver ablative doses (e.g., 8–24 Gy per fraction) across 1 to 5 sessions, SBRT has shown promising capacity for systemic immune activation (182–184). CFRT regimens result in severe radiation-induced lymphopenia due to eradication of newly primed CD8^+^ T cells as they egress from lymphoid organs, fundamentally compromising systemic antitumor immunity (4, 79, 163, 185). In contrast, SBRT maximizes the release of DAMPs while dramatically compressing the treatment window, thereby sparing the TDLNs from sustained damage and protecting the circulating lymphocyte pool from radiation-induced lymphopenia.

The PEMBRO-RT phase II trial (ClinicalTrials.gov NCT02492568) reported a higher response rate in PD-L1^–^ tumors when SBRT (3 × 8 Gy) was combined with pembrolizumab, suggesting that RT benefits patients with metastatic NSCLC (186). Additionally, SBRT has been shown to enrich IFN signaling and antigen presentation pathways in nonirradiated tumor sites, driving the TME toward a more inflamed phenotype (187). Emerging data from trials such as KEYNOTE-A18 further support the concept that radiation delivery parameters fundamentally dictate immune outcomes (188). However, the physical parameters of RT, specifically total dose and fractionation schedule, exert a nonlinear influence on immunomodulation (189). As described herein, excessively large fraction sizes may attenuate cGAS/STING signaling through TREX1 induction and promote vascular damage, hypoxia, and recruitment of immunosuppressive myeloid cells (57, 63, 80, 190). Moreover, substantial heterogeneity exists across tumor types, and current clinical evidence remains insufficient to support a universally optimal hypofractionated regimen (191). The choice of radiation modality itself can be an adaptive decision; for instance, using proton therapy to intentionally spare circulating lymphocytes and preserve systemic immune competence represents a powerful adaptive strategy, although proton therapy remains constrained by limited accessibility (192). These observations underscore the imperative for personalized fractionation strategies tailored to each patient’s tumor-immune context and concurrent systemic therapy, rather than assuming a universally optimal regimen.

Concurrently, the intentional application of spatial immunomodulation offers a mechanism to systematically reprogram the immune microenvironment and mobilize effector T cells without exacerbating systemic toxicity. Modulating radiation dose at nontumor sites offers an additional avenue for systemic immune regulation. Emerging evidence indicates that intestinal low-dose irradiation at approximately 1 Gy can enhance the crosstalk between DCs and T cells, potentially upregulating CXCR6 in exhausted effector T cells and overcoming resistance to ICIs in advanced malignancies (99, 103). Conversely, higher intestinal doses escalate T cell exhaustion and induce severe gastrointestinal toxicity, reinforcing the complex relationship between radiation dosimetry and immune function (103, 193).

### Preclinical strategies with strong rationale.

Sustaining DC activation following radiation requires targeted pharmacological interventions to overcome intrinsic resistance mechanisms. Because the immunogenicity of RT relies heavily on STING signaling, integrating STING agonists provides a rational approach to amplify cytosolic DNA sensing (44, 45). To maximize this innate activation, therapeutic expansion of the DC compartment utilizing FLT3-L prior to radiation delivery establishes a robust antigen-capturing niche (85, 194). Subsequent maturation of this expanded pool can be achieved through CD40 or OX40 agonism, which effectively overrides T cell tolerance and reinstates the cross-priming capacity of DCs (195–197). The expanded DC population within TDLNs in turn fuels the proliferation of precursor exhausted CD8^+^ T cells across both the lymphatic microenvironment and the TME (194). RT acts as a critical component of an FLT3L-based cDC1-targeting regimen by providing tumor antigen release and DC maturation signals, thereby synergizing with FLT3L and TLR3/CD40 agonists to restore responsiveness to anti–PD-L1 therapy (198).

In addition, following RT-induced antigen release, lymphatic endothelial cells establish CCL21 gradients essential for guiding CCR7^+^ antigen-presenting cells into the TDLN. The lymphangiogenic factor VEGF-C promotes the efficacy of anti–PD-1 therapy and is required for the egress of CD8^+^ T cells to the TDLN in brain tumors (199). Consequently, combining VEGF-C–targeted therapies with RT or immunotherapy is a promising strategy. However, radiation-derived VEGF-C disrupts cadherin/β-catenin junctions in lymphatic endothelial cells to promote lymphatic metastasis. Given this complexity, further investigation is urgently required (200). Notably, chemotherapy plus ivonescimab (AK112), a first-in-class bispecific antibody targeting PD-1 and VEGF, has demonstrated superior efficacy compared with anti–PD-1 monotherapy plus chemotherapy in advanced squamous NSCLC in the phase III HARMONi-6 trial ((ClinicalTrials.gov NCT05840016) (201). By simultaneously blocking the PD-1 inhibitory axis and neutralizing VEGF-mediated immunosuppression and angiogenesis, ivonescimab remodels the TME toward an inflamed, vascular-normalized state, which may be particularly amenable to combination with RT (201).

### Forward-looking hypotheses.

Enhancing stemness while mitigating exhaustion is an important strategy in cancer immunotherapy. Beyond classic TCR or costimulation, metabolic signals and TME factors (such as hormones or nucleic acid–sensing signals) further shape this state and can drive T cell differentiation toward terminal exhaustion (97, 202, 203). For instance, the ablation of prostaglandin E2 signaling to reconstitute IL-2 pathways elicits superior maintenance of TCF1^+^ T_PEX_ responses compared with direct transcriptional interference (204). Furthermore, mregDCs represent a promising therapeutic target due to their simultaneous expression of antigen-presenting and inhibitory molecules. Forward-looking strategies include bispecific constructs that bridge DCs and T cells to support CXCR6^+^CD8^+^ T cell responses and therapies selectively targeting mregDC-specific markers (e.g., LAMP3, CD200) to maximize antitumor efficacy while minimizing systemic autoimmunity (177, 205).

Beyond monitoring the TCF1^+^ progenitor pool, recent single-cell analyses have further identified GZMK^+^CD8^+^ T cells as a distinct and functionally heterogeneous subset within the TME, which express lower perforin and cytotoxic molecules, but higher proinflammatory cytokines and memory/residency markers compared with their GZMB^+^ cytotoxic counterparts (117, 206). GZMK^+^CD8^+^ T cells represent a transitional population along the differentiation trajectory toward terminal exhaustion, characterized by effector-like functions but lacking canonical exhaustion signatures (207, 208). In several cancers, the expansion of GZMK^+^ T cells correlates with better responses to immunotherapy or RT (208, 209). In contrast, in colorectal cancer, GZMK^hi^ effector memory CD8^+^ T cells interact with neutrophils to degrade E-cadherin and drive progression, predicting poor outcome (210). These context-dependent roles highlight GZMK^+^CD8^+^ T cells as promising biomarkers and therapeutic targets in RT and immunotherapy that need to be further validated in preclinical tumor models and radioimmunotherapy-responding cancer patients. Further work is also needed to elucidate their interactions with other immune cell types in TME.

### Artificial intelligence–guided radioimmunotherapy.

Building upon the biological insights and therapeutic strategies outlined above, artificial intelligence (AI) is now connecting RT precision with immune system biology by integrating comprehensive patient information, accelerating the development of optimal and individualized radioimmunotherapy strategies. For example, deep learning–based multimodal fusion frameworks extracting valuable features from radiological imaging (e.g., CT, PET, or MRI) have been shown to improve prediction of cancer treatment response (211, 212). The fusion of radiological and pathological datasets enables noninvasive virtual biopsy and achieves high-accuracy (AUC = 0.88) classification of histopathologic subtypes (213). AI is also increasingly used to identify predictive biomarkers, including signatures associated with tumor-infiltrating lymphocytes, PD-L1 expression, and immune phenotypes, thereby supporting patient stratification for radioimmunotherapy (212, 214).

AI can also be used to dynamically characterize TME. By integrating digital pathology, spatial transcriptomics, imaging, and blood-based biomarkers, AI models can quantify immune cell spatial organization, monitor treatment-induced immune remodeling, and predict therapeutic efficacy or toxicity (212). Radiomic signatures derived from AI analysis of routine CT images can serve as validated surrogates for tumor-infiltrating CD8^+^ T cell density, predicting both lesion-level response and abscopal effects in patients treated with immunotherapy and RT (215). Notably, PET radiomics can now noninvasively infer T cell exhaustion status following ablative RT (216). This real-time visualization of immune cell functional decline provides a critical biological readout and may ultimately guide the selection of optimal temporal windows for sequencing immunotherapy combinations. In addition, spatial analysis enables immunologically optimized RT, where beam trajectories are optimized to spare critical lymphocyte-rich niches. Such personalized dosimetry minimizes radiation-induced lymphopenia while maintaining strict microscopic tumor control (217). These multiscale models have provided the foundation for the lymphocyte-sparing artificial intelligence–guided radioimmunotherapy framework, which represents the first prospective clinical effort to develop a unified lymphocyte-sparing AI-guided radioimmunotherapy platform (218).

In summary, the integration of morphological, spatial, and functional multiscale imaging parameters supports predictive models for radioimmunotherapy by inferring the systemic immune landscape. However, these technologies remain emergent, and clinical evidence is sparse. Multicenter trials are essential to validate these tools in real-world practice in the future.

## Conclusion

Here, we highlighted the DC/CD8^+^ T cell axis as the core biological foundation of effective radioimmunotherapy. RT functions as a potent in situ vaccine, initiating ICD that releases tumor antigens and DAMPs, thereby priming the adaptive immune response against malignancies. However, a durable systemic response depends on the precise modulation of DCs and the preservation of stem-like CD8^+^ T cells. Importantly, beyond this central DC/CD8^+^ T cell axis, tumor-intrinsic antigen presentation competence, vascular integrity, and the broader immune infiltrate — including myeloid suppressor populations, tissue-resident memory cells, γδ T cells, and NK cells — collectively shape therapeutic outcomes.

The integration of RT and ICI has demonstrated profound potential, yet clinical outcomes exhibit striking heterogeneity. This divergence highlights a critical spatiotemporal conflict: conventional fractionated regimens and ENI inherently antagonize the systemic immune responses required for immunotherapy to succeed. Thus, future prospective studies should clarify whether combining RT with immunotherapy offers superior efficacy compared with chemo-immunotherapy regimens. Critically, these treatment platforms should integrate immunologically guided hypofractionation to minimize collateral damage to TDLNs and circulating lymphocytes.

Meanwhile, understanding mechanistic differences between different synergy strategies of ICI with RT is fundamental to advancing next-generation radioimmunotherapies with improved therapeutic outcomes. Integrating targeted biological agents — such as FLT3-L, CD40 agonists, or BiTEs — may resolve specific immunoregulatory defects within the TME. Concurrent RT generates neoantigens that provide highly specific targets for next-generation CAR T and TCR T therapies. Transcription factor editing or small molecule–based immunomodulators (such as hormone receptor degraders) that can direct engineered T cells to a resilient, stem-like state may enhance the durable antitumor response within the irradiated microenvironment. Ultimately, AI-guided radiation delivery with advanced engineered immunotherapy precision promises to overcome the spatiotemporal barriers that have limited radioimmunotherapy efficacy.

## Conflict of interest

The authors have declared that no conflict of interest exists.

## Funding support

- National Natural Science Foundation of China (U25C2023, 82350115, and 82071741 to LD; 32470962 to LL).
- Shanghai QiYuan Innovation Foundation (QY2025-GZ-001 to LD).
- Fundamental Research Funds for the Central Universities and Interdisciplinary Program of Shanghai Jiao Tong University (YG2021ZD03 to LD; YG2025QNB38 to LL).

## Version 1 08/17/2026

Electronic publication
